# Joint approach of diffusion- and perfusion-weighted MRI in intra-axial mass like lesions in clinical practice simulation

**DOI:** 10.1371/journal.pone.0202891

**Published:** 2018-09-07

**Authors:** Ra Gyoung Yoon, Ho Sung Kim, Gil Sun Hong, Ji Eun Park, Seung Chai Jung, Sang Joon Kim, Jeong Hoon Kim

**Affiliations:** 1 Department of Radiology, Eulji Medical Center, Eulji University College of Medicine, Seoul, Korea; 2 Department of Radiology and Research Institute of Radiology, University of Ulsan College of Medicine, Asan Medical Center, Seoul, Korea; 3 Department of Neurosurgery, University of Ulsan College of Medicine, Asan Medical Center, Seoul, Korea; George Washington University, UNITED STATES

## Abstract

Although advanced magnetic resonance imaging (MRI) techniques provide useful information for the differential diagnosis of intra-axial mass-like lesions, the specific diagnostic role of multimodal MRI over conventional magnetic resonance imaging (CMRI) alone in the differential diagnosis of mass-like lesions from a large heterogeneous cohort has not been studied. In this study, we aimed to determine the added value of a joint approach of diffusion-weighted imaging (DWI) and dynamic-susceptibility-contrast perfusion imaging (DSC-PWI) for diagnosis of intra-axial mass-like lesions, comparing them with CMRI alone. Furthermore, we performed these evaluations in a manner simulating clinical practice. Our institutional review board approved this retrospective study and waived the requirement for informed consent. A total of 1038 patients with intra-axial mass-like lesions were retrospectively recruited according to their histological and clinico-radiological diagnoses made between January 2005 and December 2014. All patients underwent CMRI, DWI and DSC-PWI. The diagnostic accuracy and confidence in diagnosing each type of intra-axial mass-like lesions, and for differentiating the intra-axial brain tumors from non-neoplastic lesions, were compared according to the MRI protocols. The disease-specific sensitivity of joint approach differed according to specific disease entities in diagnosing each disease category. Joint approach provided the best diagnostic accuracy for discriminating intra-axial brain tumors from non-neoplastic lesions, with high diagnostic accuracy (95.3–96.7%), specificity (82–84.0%), positive-predictive-value (97.0–97.3%), and negative-predictive-value (84.8–92.7%), with the reader’s confidence values being significantly improved over those on CMRI alone (all *p*-values < 0.001). In conclusion, joint approach of DWI, DSC-PWI to CMRI helps to differentiate non-neoplastic lesions from intra-axial brain tumors, and improves diagnostic confidence compared with CMRI alone. The benefit from the combined imaging differs for each disease category; thus joint approach needs to be customized according to clinical suspicion.

## Introduction

During recent decades, advanced magnetic resonance imaging (MRI) techniques, such as diffusion-weighted imaging (DWI), dynamic susceptibility contrast perfusion imaging (DSC-PWI), and magnetic resonance spectroscopy (MRS) have become available as adjuncts to conventional magnetic resonance imaging (CMRI) techniques have increased diagnostic accuracy in the differentiation of intra-axial brain tumors [1–5]. These MRI protocols can noninvasively reflect the pathophysiological characteristics of brain tumors, and have thus been incorporated into routine protocols for evaluating patients with intra-axial mass-like lesions (MLLs). Notably, DSC imaging provides hemodynamic information associated with increased microvascular density in brain tumors, inflammatory activity, and vascular compromise in inflammatory lesions [6]. DWI imaging can reflect changes in cellularity in various brain tumors, and improves the specificity of MRI for distinguishing brain abscesses and cystic tumors [7].

Although each advanced MRI technique provides useful information for the differential diagnosis of intra-axial MLLs, most previous studies have focused on the diagnostic ability of a single imaging technique [8–11], rather than comprehensive evaluation of the diagnostic value of a multimodal MRI protocol including DWI and DSC-PWI. Furthermore, many previous studies have performed quantitative analysis of separate MRI sequences and presented threshold values, whereas in clinical practice, radiologists tend to read each MRI exam through visual assessment, combining the information from various sequences. Moreover, the specified diagnostic roles of a protocol including such multiple MRI sequences in the differential diagnosis of all-encompassing inflammatory lesions such as tumefactive demyelinating lesions or abscess and tumorous conditions such as lymphoma, metastasis, or gliomas in heterogeneous cohorts have not been studied.

As a tertiary referral hospital, we have routinely applied a single-session multimodal MRI protocol, including DWI and DSC-PWI in addition to CMRI to patients with intra-axial MLLs. In this study, we sought to evaluate the diagnostic role and influence on diagnostic pattern of this joint approach of DWI and DSC-PWI compared to CMRI alone. In this evaluation, we tried to simulate clinical practice by including patients presenting with any intra-axial MLLs, thereby including a wide range of disease entities.

## Materials and methods

### Patient population

The protocol of this retrospective study was approved by the institutional review board of Asan Medical Cetner and the need for informed consent was waived [http://eirb.amc.seoul.kr]. A retrospective review of our institution’s database for the period January 2005 to December 2014 identified 4,981 patients who had undergone an MRI protocol including conventional MRI, DWI, and DSC-PWI for the evaluation of intra-axial MLLs. MLLs were defined as volumetric space-occupying lesions distinct from normal-appearing brain parenchyma on conventional MRI. The following exclusion criteria were applied patients with an extra-axial brain mass; those younger than 18 years of age; those who had undergone surgery or treatment, i.e., steroid therapy or radiation, before the MRI examination; those either a follow-up MRI for clinical diagnosis, or histologic diagnosis; and those without complete MRI protocols. Finally, 1,038 patients (mean age ± SD, 50.6 ± 14.9 years) who initially presented with intra-axial MLLs were included in our study. Among these 1038 patients, 150 were diagnosed with non-neoplastic lesions and 888 with intra-axial brain tumors. The 150 patients with non-neoplastic lesions included 116 classified as non-neoplastic other brain diseases (NOBDs; 30 demyelinating disease, 32 subacute or venous infarction, 22 encephalitis, 17 vasculitis, six traumatic contusion, one hippocampal sclerosis), 21 classified as abscess, and 13 classified as tumefactive demyelinating disease (TDL). The NOBDs were defined as non-neoplastic mass-like brain lesions other than TDL and abscess. Among the 888 patients with tumors, 92 had lymphoma, 210 had low-grade glioma (LGG), 127 had metastasis, 368 had high-grade glioma (HGG), and 91 were diagnosed with other brain tumors. The World Health Organization neoplasm grading scheme was employed for intra-axial brain tumors.

### Reference standard for the final diagnosis

The final diagnosis was made from either pathologic report (surgical resection or biopsy), CSF cytology analysis (i.e. encephalitis, lymphoma), or clinico-radiologic diagnosis. For clinico-radiologic diagnosis, a follow-up MRI was used as the reference standard. For intra-axial brain tumors, the World Health Organization neoplasm grading scheme was employed in the pathologic report. In the 150 patients with non-neoplastic lesions, 51 patients were histologically confirmed, and 99 patients were clinico-radiologically diagnosed. Of these 99 patients, 32 patients with infarction were confirmed by angiography, 22 with encephalitis were confirmed by CSF analysis and a positive response to antibiotics, 17 with vasculitis were confirmed by laboratory findings and a positive response to steroid therapy, six patients with traumatic contusion were confirmed by trauma history and follow-up MRI, three patients with demyelinating disease and nine patients with TDL were confirmed by follow-up MRI and a positive response to steroid therapy, and one patient with hippocampus sclerosis was confirmed history of recurrent seizure, electroencephalography, and follow-up MRI [12]. Of the 888 patients with intra-axial brain tumors, 745 patients were histologically confirmed, and 143 patients were clinico-radiologically diagnosed. Of the 143 clinico-radiologically diagnosed patients, six with lymphoma were confirmed by cytology analysis [13,14], 51 with metastasis were confirmed by the identification of a primary neoplasm in another organ with multiple metastasis and/or additional brain metastasis on follow-up MRI [12], 64 with LGG were confirmed by MR imaging findings and long-term follow-up MRI (mean time, 1049 ± 757.4 days), and ten with HGG were confirmed by agreement between the neurosurgeon and the neuroradiologist after complete review of both the clinical information and the MR imaging findings, with no identification of malignancy in other organs during the follow-up period (mean time, 314.7 ± 140.7 days). The remaining 12 patients with other brain tumors were diagnosed by long-term follow-up MRI after exclusion of the aforementioned brain tumors (mean time, 1194.3 ± 1026.3 days).

### Imaging technique

CMRI, DWI, and DSC-PWI were performed using a 3T system (Achieva; Philips Medical Systems, Best, The Netherlands) with an eight-channel, sensitivity-encoding head coil. The imaging protocol was acquired in the following order: T2-weighted imaging, DWI, T1-weighted imaging, gadolinium-enhanced T1-weighted imaging, and DSC-PWI. DWI was acquired in three orthogonal directions and combined into a trace image. Using this data, i.e. b-values of 0 and 1000 s/mm2 on a voxel-by-voxel basis, the software incorporated into the MR imaging unit calculated the apparent diffusion coefficient (ADC) map. The DWI parameters were as follows: repetition time (TR)/echo time (TE), 3000/56 ms; field of view (FOV), 25 cm; slice thickness/gap, 5 mm/2 mm; matrix, 256 x 256; diffusion gradient encoding, b = 0, and 1000 s/mm2; and acquisition time, 39 seconds. DSC-PWI was performed using a gradient-echo, echo-planar sequence during which a standard dose of 0.1 mmol/kg of gadoterate meglumine (Dotarem; Guerbet, Paris, France) was administered at a rate of 4 mL/s using an MR-imaging-compatible power injector (Spectris; Medrad, Pittsburgh, PA, USA). The bolus of contrast material was followed by a 20-mL bolus of saline administered at the same injection rate. The imaging parameters for DSC-PWI were as follows: TR/TE, 1808/40 ms; FOV, 24 cm; slice thickness/gap, 5 mm/2 mm; matrix, 128 x 128; and flip angle, 35°. The total acquisition time for DSC-PWI was 114 seconds. DSC-PWI was performed using the same section orientations as the CMRI, and covered the entire tumor volume.

### Imaging analysis

Two expert radiologists (G.S.H. and R.G.Y. with 7 and 6 years of clinical experience in brain MRI, respectively) who were blinded to the patients’ diagnosis and other clinical information, independently performed visual, semi-quantitative assessment in three reading sessions separated by an interval of over a month so as to avoid recall bias. The readers interpreted only the CMRI protocol (i.e., T2-weighted images, T1-weighted images, and gadolinium-enhanced T1-weighted images) in the first reading session, joint approach protocol of CMRI and DWI in the second reading session, and then joint approach protocol includes CMRI and DWI plus DSC-PWI in the third reading session. In each reading session, the readers interpreted all 1038 cases in a re-randomized order, suggesting up to three differential diagnoses in order of confidence from a list of the above eight disease categories, and rated their confidence level for each diagnosis using a hierarchical three-point scale: grade 3 = definite, confidence level ≥ 95%; grade 2 = probable, confidence level 70–95%; and grade 1 = possible, less than 70% confidence [15,16]. For semi-quantitative visual assessment, the ADC values were classified according to a four-point scale: ADC value < white matter (Grade 4); ADC value = white matter (Grade 3); CSF < ADC value < white matter (Grade 2); and ADC value = CSF (Grade 1) [17–19]. The following four-point grading system was used for DSC-PWI: perfusion value = vessel (Grade 4); gray matter < perfusion < vessel (Grade 3); perfusion = gray matter (Grade 2); and perfusion < gray matter (Grade 1). All MR images were assessed using a local PACS monitor and digital imaging and communications in medicine (DICOM) image viewing software (PetaVision: Asan Medical Center, Seoul, Korea).

### Analysis of diagnostic confidence

The readers’ diagnostic confidence was determined qualitatively according to a combination of confidence score from at least one to three for each differential diagnoses, and the number of diagnoses made for each case. The highest reader confidence was considering one diagnosis with grade 3 confidence, and the lowest reader confidence was considering three differential diagnoses with all grade 3 confidence. The reader confidence was scored from 1 to 9 points with 9 being the highest score as follows: 1 point = three differential diagnoses all with grade 3 confidence; 2 points = three differential diagnoses two with grade 3 and one with grade 2 confidence; 3 points = three differential diagnoses, two with grade 3 and one with grade 1 confidence; 4 points = two differential diagnoses, both with two grade 3 confidence); 5 points = three differential diagnoses, one with grade 3 and two with grade 2 confidence; 6 points = three differential diagnoses, one with grade 3, one with grade 2, and one with grade 1 confidence; 7 points = two differential diagnoses, one with grade 3 and one with grade 2 confidence; 8 points = two differential diagnoses, one with grade 3 and one with grade 1 confidence; and 9 points = one diagnosis with grade 3 confidence. The diagnostic confidence values are illustrated using the heat maps [20–22].

### Statistical analysis

The overall diagnostic accuracies of the three MRI protocol sets for all intra-axial MLLs were compared using logistic regression. A decision threshold of grade 3 was considered positive, and the diagnostic accuracy, sensitivity, specificity, positive predictive value (PPV), and negative predictive value (NPV) for the discrimination of neoplastic lesions from non-neoplastic lesions, and the per-disease sensitivity and PPV for diagnosis of each disease entity, were compared between the three MRI protocols using logistic regression with generalized estimating equations. The generalized estimating equation models by eight diseases with a diagnostic value of Yes or No for each disease as the dependent variable, and the (CMRI vs CMRI and DWI vs. CMRI and DWI and DSC-PWI) as independent variables. The correlation structure was treated as an exchangeable working correlation structure. The diagnostic confidence values for diagnosis of intra-axial MLLs were compared between MRI protocols using a paired *t*-test. The complete data on diagnostic confidence are presented using heat maps [17, 18, 20]. Interobserver agreements in diagnosing intra-axial MLLs were measured using kappa statistics. A *p*-value < 0.05 was considered statistically significant for overall comparisons between the imaging protocol sets.

## Results

### Summary of patient characteristics and clinical information

Of the 1038 patients, 150 were diagnosed with non-neoplastic lesions and 888 with intra-axial brain tumors. The demographic characteristics and clinical information of the study patients are summarized in Table 1.

**Table 1:** Patient characteristics and clinical information for All intra-axial mass-like lesions.

Characteristics	Non-neoplastic lesions				Neoplasms				
	All patients	NOBD	Abscess	TDL	Lymphoma	LGG	Metastasis	HGG	OBT
No. of patients	1038	116	21	13	92	210	127	368	91
Sex (M/F)	591/447	64/52	16/5	5/8	59/33	122/88	79/48	204/164	42/49
Age, years (Mean±SD)	50.6±14.9	48.8±15.7	55.9±12.5	42.8±13.0	58.7±12.7	42.4±12.9	58.5±10.4	53.8±13.2	40±17.1
Surgery or biopsy	796	26	21	4	86	146	76	358	79
Interval between MRI and surgery in days^a^	17.0±27.4(0–261)	43.3±79.8(0–261)	5.0±4.8(0–18)	13.8±.8(9–18)	8.6±9.7(0–52)	33.1±39.6(0–218)	10.2±14.3(1–31)	11.4±14.4(1–65)	20.8±20.4(1–94)
Clinicoradiologic diagnosis	242	90	0	9	6	64	51	10	12
Interval between initial and follow-up MRIs in days^a^	630.1±666.1(18–4042)	529.5±546.7(18–2618)		886.8±679.3(366–2294)	477.2± 273(260–904)	1049.2±757.4(366–2746)	183.6±101.1(35–362)	314.7±140.7(156–557)	1194.3±1026.3(370–4042)

NOBDs = non-neoplastic other brain disease; TDL = tumefactive demyelinating lesion; LGG = low-grade glioma; HGG = high-grade glioma; OBT = other brain tumor; MRI = magnetic resonance imaging; SD = standard deviation.

^a^ Data are mean ± standard deviation with range in parentheses.

### Added value of joint approach MRI protocols for discriminating neoplasms from non-neoplastic lesions

Joint approach of CMRI with DWI plus DSC-PWI improved overall diagnostic accuracy in comparison with CMRI alone (84.3% versus 80.0% for reader 1, 82.7% versus 78.0% for reader 2) (all *p*-values < 0.001). Compared with CMRI alone, joint approach showed increased accuracy (96.7% for reader 1, 95.3% for reader 2), specificity (84.0% for reader 1, 82.0% for reader 2), PPV (97.3% for reader 1, 97.0% for reader 2) and NPV (92.7% for reader 1, 84.8% for reader 2) in the discrimination of neoplasms from non-neoplastic lesions. Of particular note is that the joint approach increased specificity by 10.7% for reader 1 and 9.3% for reader 2. Reader2 showed slightly decreased sensitivity using the joint approach (97.5%) in comparison with CMRI alone (97.8%). The kappa values between the two readers were 0.716–0.749 for CMRI alone and 0.781–0.801 for joint approach. Table 2 summarizes the differences in diagnostic performance between the joint approach protocol and CMRI alone for the discrimination of neoplastic from non-neoplastic lesions.

**Table 2:** Diagnostic performance of joint approach protocols and conventional MRI alone in discriminating neoplasms from non-neoplastic lesions.

MRI Protocols	Accuracy,%(95% CI)	Sensitivity,%(95% CI)	Specificity,%(95% CI)	PPV,%(95% CI)	NPV,%(95% CI)
MRI alone					
-Reader 1	95.0(93.5–96.2)	98.7(97.7–99.2)	73.3(65.7–79.8)	95.6(94.1–96.8)	90.2(83.6–94.3)
-Reader 2	94.1(92.5–95.4)	97.8(96.6–98.5)	72.7(65.0–79.2)	95.5(93.9–96.7)	84.5(77.3–89.7)
Joint approach of C+D					
-Reader 1	95.9(94.5–96.9)	98.6(97.7–99.2)	79.3(72.2–85.0)	96.5(95.2–97.6)	90.8(84.7–94.7)
-Reader 2	94.9(93.4–96.1)	97.3(96.0–98.2)	80.7(73.6–86.2)	96.8(95.4–97.7)	83.5(76.6–88.6)
Joint approach of C+D+P					
-Reader 1	96.7(95.5–97.7)	98.9(97.9–99.4)	84.0(77.3–89.0)	97.3(96.1–98.2)	92.7(87.0–96.0)
-Reader 2	95.3(93.8–96.4)	97.5(96.3–98.4)	82.0(75.1–87.3)	97.0(95.6–97.9)	84.8(78.1–89.8)

CMRI = conventional magnetic resonance imaging; C, conventional magnetic resonance imaging; D, diffusion-weighed imaging; P, dynamic susceptibility contrast perfusion imaging

### Comparison of disease-specific sensitivities and PPVs

The disease-specific sensitivity, PPV and inter-observer agreement for the diagnosis of intra-axial MLLs are shown in Table 3 for the combined protocol and CMRI alone. For diagnosis of NOBDs, the joint approach significantly improved disease-specific sensitivity in comparison with CMRI alone (from 66.4% to 78.5% for reader 1, from 64.7% to 76.7% for reader 2, *p*-value = 0.003 and 0.008, respectively). For diagnosis of brain abscess, the joint approach protocols significantly improved sensitivity (from 66.7% to 100%) and PPV for reader2 (from 82.4% to 91.3% for reader 1, from 73.7% to 91.3% for reader 2, *p*-value = 0.064 and 0.006, respectively). However, for TDL, the sensitivity of joint approach did not differ significantly from CMRI alone for either readers (*p*-value = 0.717 and 1, respectively), and the PPV decreased for reader 1 (from 91.7% to 68.8%, *p*-value = 0.275). In the diagnosis of intra-axial brain tumors, the joint approach showed higher sensitivity and PPV for lymphoma (81.5% to 87.0%, and 84.3% to 86% for reader 1, 75% to 89.1% and 80.2% to 89.1% for reader 2, *p*-value = 0.001 and 0.168 for sensitivity, *p*-value = 0.675 and 0.009 for PPV, respectively), metastasis (89% to 89.8% and 76.9% to 77% for reader 1 and 87.4% to 89.8% and 67.3% to 74% for reader 2, *p*-value = 0.077 and 0.382 for sensitivity, *p*-value = 0.108 and 0.008 for PPV, respectively), and HGG (87.5% to 85.9% and 85.9% to 86.3% for reader 1, 84% to 85.3% and 86.1% to 87.2% for reader 2, *p*-value = 0.133 and 0.139 for sensitivity, *p*-value = 0.310 and 0.598 for PPV). In addition, the joint approach showed higher sensitivity for LGG (77.1% to 83.3% for reader 1, *p*-value = 0.012). For the diagnosis of other brain tumors, the joint approach showed significantly improved sensitivity and PPV (64.8% to. 73.6% and 72.0% to 85.9% for reader 1, 56.0% to 74.7% and 67.1% to 87.2% for reader 2, *p*-value = 0.021 and < 0.001 for sensitivity, *p*-value < 0.001 for PPV, respectively).

**Table 3:** Comparison of disease-specific sensitivity and PPV between the joint approach protocols and conventional MRI alone.

	Non-neoplastic Lesions						Neoplasms									
	NOBDs		Abscess		TDL		Lymphoma		LGG		Metastasis		HGG		OBT	
MRI protocols	R1	R2	R1	R2	R1	R2	R1	R2	R1	R2	R1	R2	R1	R2	R1	R2
CMRI alone																
Sen, %	66.4	64.7	66.7	66.7	84.6	61.5	81.5	75.0	77.1	80.0	89.0	87.4	87.5	84.0	64.8	56.0
PPV, %	89.5	85.2	82.4	73.7	91.7	42.1	84.3	80.2	90.0	88.0	76.9	67.3	85.9	86.1	72.0	67.1
k, [SE]	0.701 [0.058]		0.830 **[**0.109]		0.388 [0.218]		0.603 [0.089]		0.674 [0.056]		0.773 [0.080]		0.738 [0.047]		0.623 [0.071]	
Joint approach of C + D																
Sen, %	70.7	74.1	100	100	84.6	69.2	87.0	83.7	82.9	79.5	85.8	85.8	84.5	82.3	69.2	65.9
PPV, %	91.1	83.5	80.8	77.8	78.6	69.2	84.2	86.5	82.9	79.9	73.2	72.2	84.3	85.6	81.8	77.9
k, [SE]	0.750 [0.060]		*		0.618 [0.232]		0.762 [0.093]		0.837 [0.044]		0.665 [0.089]		0.751 [0.042]		0.743 [0.065]	
Joint approach of C + D + P																
Sen, %	78.5†	76.7†	100 †	100†	84.6	61.5	87.0	89.1†	83.3†	77.1	89.8	89.8	85.9	85.3	73.6†	74.7†
PPV, %	93.8	80.9	91.3	91.3†	68.8	66.7	86.0	89.1†	83.7	80.2†	77.0	74.0†	86.3	87.2	85.9†	87.2†
k, [SE]	0.720 [0.072]		*		0.422 [0.206]		0.864 [0.074]		0.704 [0.058]		0.804 [0.082]		0.802 [0.042]		0.704 [0.074]	

* kappa value is not available because of the 100% inter-observer agreement

† Statistically significant with a *p*-value less than 0.05.

CMRI = conventional magnetic resonance imaging; Joint approach = combination of CMRI, diffusion-weighed imaging, and dynamic susceptibility contrast magnetic resonance perfusion imaging; NOBDs = non-neoplastic other brain diseases; TDL = tumefactive demyelinating lesion; LGG = low-grade glioma; HGG = high-grade glioma; MLL = mass-like lesions; Sen = sensitivity; PPV = positive predictive value. Logistic regression using generalized estimating equations was used for the between protocol comparisons of the disease-specific sensitivity and PPV.

### Diagnostic confidence in discrimination of non-neoplastic lesions from neoplasms on different imaging protocols

**Fig 1:**
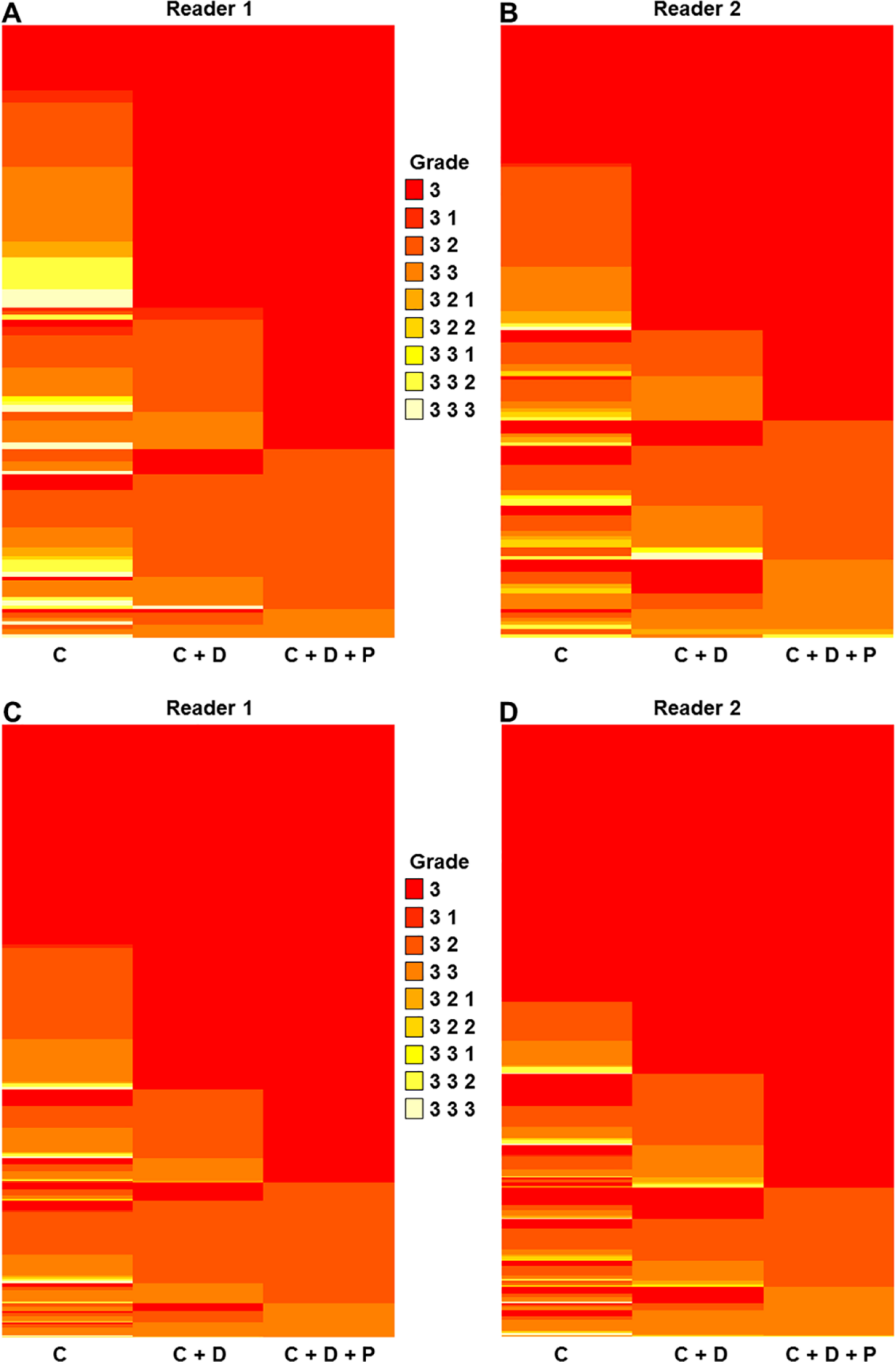
Heat maps of diagnostic confidence for intra-axial non-neoplastic lesions and neoplasm groups. (A-B) Heat maps for 150 intra-axial non-neoplastic lesions. (C-D) Heat maps for 888 neoplasms. At each intersection in the maps, the grid element color represents the grade assigned by the MRI protocols (i.e., conventional imaging, joint approach of DWI and CMRI, and joint approach of DWI and DSC-PWI and CMRI; columns) and the corresponding cases (rows). The confidence grades 1 to 9 were mapped with colors from light yellow to dark red. In the same manner, two heat maps were drawn for each reader for each of the non-neoplastic lesions and neoplasm groups. A dark red color indicates a high confidence grade, and a light yellow color indicates a lower confidence grade. C = Conventional MRI; C+D = joint approach combining conventional MRI, diffusion-weighed imaging; C+D+P = joint approach combining conventional MRI, diffusion-weighed imaging, and dynamic susceptibility contrast perfusion imaging.

All data concerning diagnostic confidence are summarized in Table 4 and illustrated in Fig 1. Compared with CMRI alone, the joint approach protocols significantly improved the confidence of all readers for diagnosing non-neoplastic lesions (*p*-values < 0.001), and intra-axial brain tumors (*p*-values < 0.001). In comparison with those from CMRI, the combined approach heat maps demonstrated an increase in the areas representing higher confidence levels (i.e., dark red color) for the diagnosis of non-neoplastic lesions and intra-axial brain tumors, and a decrease in the areas representing lower confidence levels (i.e., light red and yellow colors).

**Table 4:** Readers' confidence ratings for the different imaging protocols.

Grade	Non-neoplastic Lesions						Neoplasms					
	R1 (p value <0.001)			R2 (p value <0.001)			R1 (p value <0.001)			R2 (p value <0.001)		
	C	C+D	C+D+P	C	C+D	C+D+P	C	C+D	C+D+P	C	C+D	C+D+P
3	24	76	104	52	89	97	387	567	664	537	577	671
31.	6	3		1			6			5		
32.	41	51	39	51	30	34	264	237	175	180	174	144
33.	43	19	7	23	27	17	187	80	49	116	111	70
321	6			8	1	1	8	4		6	14	1
322	1			5			7			17	5	1
331	1			1	1						1	
332	13			8		1	18			18	4	
333	13			1	2		11			9	2	1

R1, reader 1; R2, reader 2; C, conventional magnetic resonance imaging; D, diffusion-weighed imaging; P, dynamic susceptibility contrast magnetic resonance perfusion imaging

## Discussion

In this study, we evaluated the added value of joint approach of DWI and DSC-PWI over CMRI alone for diagnosing intra-axial MLLs at initial presentation, performing the image interpretation in a manner simulating clinical practice. We included a large number of patients diagnosed with various intra-axial MLLs and attempted to simulate the diagnostic approach used in clinical practice by recording differential diagnosis lists in order of confidence. The joint approach protocols of CMRI with DWI and DWI plus DSC-PWI improved specificity for differentiation of non-neoplastic lesion from intra-axial brain tumors, with a significant improvement in the diagnostic confidence of the expert readers. However, the impact of the joint approach was not uniform, but differed according to the specific disease. For intra-axial brain tumors, the joint approach protocol improved sensitivity and PPVs for all tumors except low grade glioma, and showed the most improvement for lymphoma. For non-neoplastic lesions, joint approach significantly improved the differentiation of abscess and NOBDs, achieving 100% sensitivity for abscesses. However, for TDLs, both the sensitivity and PPV were lower with the joint approach protocols than with CMRI, and the kappa value was the lowest among the MLLs. Thus, our findings support the clinical use of joint approach of DWI and DSC-PWI in patients with intra-axial MLLs, indicating that it has added value for differentiating non-neoplastic lesions and for narrowing-down specific disease entities. However, considering the modest improvement in diagnostic accuracy achieved with the joint approach, we consider that the added value of these joint approach protocols over CMRI alone is perhaps insufficient to justify their standard use in initial routine evaluations of most intra-axial MLLs. CMRI alone would minimize unnecessary additional cost for the initial evaluation, and then customized combined protocols could be used for those patients requiring them. This would ensure accurate stratification of patients for needing just follow-up imaging or surgical resection or medical treatment.

A potential strength of this study is that we evaluated the actual diagnostic performance of the joint approach MRI protocols in the diagnosis of various intra-axial MLLs using a method simulating that used in clinical practice. Although there have been a number of reports seeking to define the diagnostic role of DWI or DSC MRI, they are generally concerned with only two or three disease categories [1, 11, 23–28]. This does not reproduce the actual diagnostic decision making encountered in daily clinical practice, where decisions are made between various differential diagnositc lists. Here, this study included 1038 patients who initially presented in our hospital, and we tried to determine the diagnostic role of joint approach in clinical practice using visual analysis of expert readers and recording differential lists to simulate a radiologic report. Thus, our study provides comprehensive information on the practical diagnostic performance and effect on diagnostic confidence of varying combinations of DWI, DSC-PWI added to CMRI for diagnosis of intra-axial MLLs.

In terms of diagnostic performance, we found that the added value of joint approach was improved diagnostic accuracy, sensitivity, specificity, PPV, and NPV for differentiating neoplasm from non-neoplastic lesions, while DWI provided a definite benefit for diagnosing brain abscesses and lymphoma. DWI shows high signal where the motion of water within tissue is restricted [1, 11, 26–28], and this enabled the readers to differentiate abscess and lymphoma from other MLLs according to the areas with restricted diffusion. Moreover, when joint approach of CMRI with DWI was applied, the specificity for discriminating neoplasms from non-neoplastic lesion was the highest, above that of joint approach of DWI and DSC-PWI. Therefore, considering the superior diagnostic information and relatively short acquisition time of DWI, it can be claimed that the joint approach of CRMI with DWI could be used as a routine evaluation protocol for screening of intra-axial MLL patients. However, for TDL, the joint approach reduced the PPV for reader 1 compared with CMRI alone, and there was no significant difference of diagnostic sensitivity. Although this may be related to the low incidence and nonspecific imaging characteristics of TDLs, which sometimes mimic neoplastic lesions such as glioblastoma or lymphoma [29], it may also be caused by misclassification to other disease categories by misreading of the variety of information presented on the advanced MRI sequences. Another possible explanation could be our use of semi-quantitative visual assessment without measurement of the absolute ADC value and relative cerebral blood volume (rCBV).

For most intra-axial brain tumors, the joint approach improved sensitivity and PPV. For lymphoma, metastases, and other brain tumors, both the sensitivity and PPV increased by 4% to 9% with the addition of DWI and DSC-PWI, and for most tumors the improvements were statistically significant. However, the increases in sensitivity and PPV for LGG and HGG were only around 1%, and did not reach statistical significance. These results may imply that the information obatined from DSC-PWI could help to differentiate specific types of neoplasms, but that it would not be necessary to add DWI or DSC imaging to CMRI to make a differential diagnosis for grading of brain gliomas; for these, the infiltrative component and contrast-enhancing portion can be sufficiently assessed with conventional MRI. However, the value of quantitative assessments of ADC value and rCBV as prognostic and predictive factors is not a main subject of interest in this study, which cannot be offered with semi-quantitative visual assessment on CMRI alone [19, 30, 31].

The joint approach significantly improved the diagnostic confidence over CMRI alone in all entities of intra-axial MLLs. Furthermore, the joint approach protocols of CMRI with DWI and DWI plus DSC-PWI led to higher confidence in the diagnosis of non-neoplastic lesions and neoplasms compared with just DWI and CMRI. In clinical practice, the radiologic diagnostic approach for intra-axial MLLs is usually based on a hierarchical structure with stratification into categories of diagnostic certainty or confidence among the various diagnostic lists, contrary to a study setting in which a researcher selects one diagnosis. The higher certainty for intra-axial MLL diagnosis with the joint approach protocol could narrow the differential diagnosis and have clinical value for the next phase of decision-making and further examination choices. However, whether an improvement in diagnostic confidence will result in better clinical outcomes remains unknown.

Our study had several limitations. Its retrospective design may have introduced case selection bias, as the study population consisted of patients at a single tertiary care center who presented with intra-axial MLLs. However, in reality, it is very difficult to perform prospective studies covering the whole sequences of advanced MRI for all types of intra-axial MLLs. In addition, in clinical practice, it is not always necessary to perform a joint approach protocol to differentiate non-neoplastic intra-axial MLLs such as infection and acute infarct. Second, this study included some patients who were diagnosed on the basis of our clinico-radiological diagnosis. However, as described above, we applied strict inclusion criteria to this clinico-radiological diagnosis. As histological confirmation for most benign brain diseases is usually difficult, we included only those patients without any change suggestive of malignant transformation at long-term follow-up (mean time interval ± SD, 183.6 ± 101.1–1194.3 ± 1026.3 days) for benign tumors. Lastly, we analyzed DSC-PWI using semi-quantitative visual assessment without measurement of the absolute ADC value and rCBV. However, our intention was to simulate clinical practice, using semi-quantitative visual assessment as is common in daily practice.

In conclusion, the joint approach of combining diffusion-and perfusion-weighted imaging in addition to conventional MRI helps to differentiate non-neoplastic lesions from intra-axial brain tumors, and improves diagnostic confidence compared with CMRI alone. The degree of benefit differs between disease categories; therefore, a joint approach needs to be customized according to clinical suspicion for intra-axial mass-like lesions.

## Supporting information

S1 File (XLSX)

S2 File (XLSX)

S3 File (XLSX)

S4 File (XLSX)
